# High expression of matrix metalloproteinases 16 is associated with the aggressive malignant behavior and poor survival outcome in colorectal carcinoma

**DOI:** 10.1038/srep46531

**Published:** 2017-04-19

**Authors:** Shengwen Wu, Congchao Ma, Shaoyin Shan, Lei Zhou, Wenhui Li

**Affiliations:** 1 Department of General Surgery, The Affiliated Jianhu Hospital of Nantong University, Jianhu People’s Hospital, Jianhu, Jiangsu Province, 224700, China; 2 Department of Interventional Radiology, The Affiliated Yancheng Hospital of Southeast University Medical College, Yancheng Third People’s Hospital, Yancheng, Jiangsu Province, 224001, China

## Abstract

Recent evidence suggested an important role of matrix metalloproteinases 16 (MMP16) in the progression of several cancers. However, the contribution of MMP16 to colorectal cancer (CRC) remains elusive. In this study, we combined analyzed the MMP16 expression in The Cancer Genome Atlas (TCGA), GSE39582 database and in-house database. In TCGA and GSE39584 database, the log-rank test demonstrated that overall survival (OS) for patients with low MMP16 expression in tumor tissues was significantly higher than those with high expression (*P* < 0.05). In the validation cohort, high MMP16 expression was significantly correlated with N stage (*P* = 0.008) and lymphovascular invasion (*P* = 0.002). The 5-year OS and disease free survival (DFS) in high and low MMP16 expression groups were 66.0% and 80.6%, 54.3% and 72.8%, respectively. Univariate and multivariate analysis showed that high MMP16 expression was an independently prognosis factor for both OS and DFS (*P* < 0.05). Functional study found that silencing MMP16 expression could inhibit migration and invasion of colon cancer cells. In conclusion, high expression of MMP16 is associated with the aggressive malignant behavior and poor survival outcome of CRC patients. MMP16 can serve as an indicator of prognosis as well as a potential novel target for treatment of CRC patients.

Globally, colorectal cancer (CRC) is the third most frequently diagnosed cancer and one of the leading causes of cancer deaths^1^. Its incidence has been increasing in China in recent years. Although most patients at early stage can be successfully cured with surgery, about 20–45% of patients who underwent curative resection developed recurrence or metastasis^2^,^3^. Currently, tumor-node-metastasis (TNM) stage is the most powerful predictor for survival in CRC. However, the survival outcome is quite different even for patients at same TNM stage. Further understanding on the biological mechanisms of the metastasis and progression of CRC and developing effective measures to target this process are of vital importance. Much attention has been focused on the molecular-based prognostic markers, which are complementary to the data obtained by pathological diagnosis and can be used to give more information for clinical practice^4^,^5^,^6^.

Matrix metalloproteinases (MMPs) are a family of zinc dependent proteases capable of degrading most extracellular matrices (ECMs). MMPs participate in many physical and pathological processes such as morphogenesis, wound healing, tissue repair, and remodeling^7^. In addition, MMPs play a critical role in tumor progression through ECM turnover and cancer-cell migration, as well as regulating signaling pathways that control cell growth, inflammation, or angiogenesis^8^,^9^. As an important member of the MMPs family, MMP16 can also exhibit proteolytic activity against components of the extracellular matrix. MMP16 is frequently overexpressed in various human cancer tissues and help facilitate cancer metastasis and progression^10^,^11^,^12^,^13^. However, the clinical significance of MMP16 expression in CRC has rarely been investigated until now.

In the present study, we analyzed the MMP16 expression levels in public available databases, The Cancer Genome Atlas (TCGA) and GSE39582 in Gene Expression Omnibus (GEO), and then validated the results in in-house database to evaluate the correlations between the MMP16 level and clinicopathological features and survival outcomes. Functional studies were also conducted to figure out the role of MMP16 in oncogenesis.

## Results

### MMP16 expression in TCGA and GSE39582 database

A total of 579 eligible patients with CRC met the selection criteria in TCGA database, including 316 males and 263 females. The median age for all patients was 66 years (rang 31–90 years old). 987.9% (509/579) patients were at M0 stage. The median length of follow-up was 25 months (range, 0–142 months) and 123 (21.2%) patients had died at the end of follow-up. Table 1 showed the baseline characteristics of the two study cohorts.

**Figure 1:**
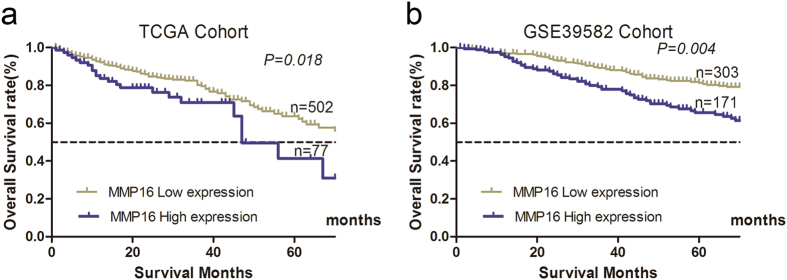
Increased MMP16 expression was significantly associated with the overall survival of CRC patients in TCGA and GSE39582 database. The data were analyzed using Kaplan-Meier survival analysis between patients with high MMP16 expression and low MMP16 expression in TCGA (**a**) and GSE39582 database.

We then divided the patients in TCGA cohort into low or high risk subgroups according to the optimal cutoff value determined by ROC curve in terms of MMP16 expression levels. The log-rank test demonstrated that OS for patients with low MMP16 expression in tumor tissue was significantly higher than those in high group (P = 0.018; Fig. 1a). Then, we validated the results in GSE39582 database, the MMP16 was further confirmed as prognostic factor (P = 0.004, Fig. 1b).

### Validation of MMP16 expression in in-house database

There were 192 eligible patients in the validation database, including 99 (51.6%) males and 93 (48.4%) females. The median follow-up period was 61 (12–89) months. Patient demographics and pathological features are summarized in Table 1.

**Figure 2:**
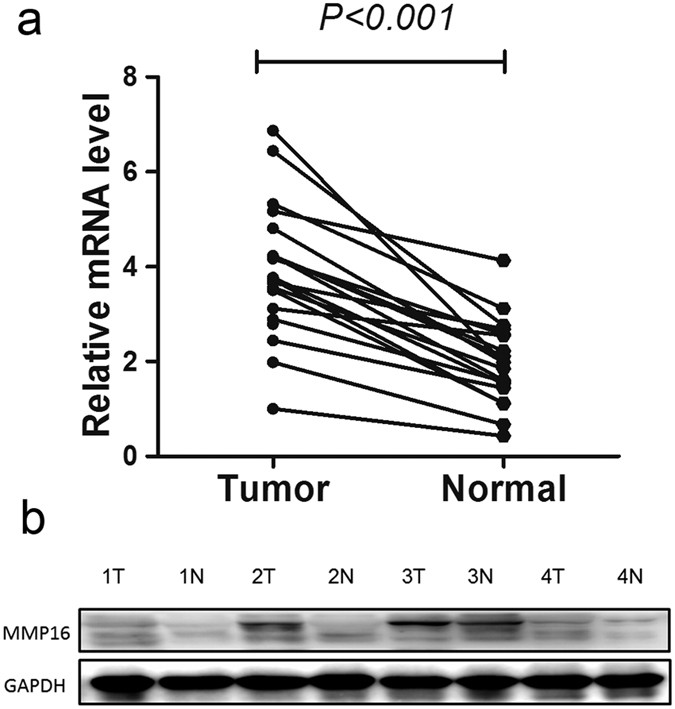
Expression of MMP16 mRNA in colon cancer tumors and adjacent normal mucosa. (**a**) Relative MMP16 mRNA levels in 20 matched colorectal tumors compared with the levels in normal mucosa specimens. The relative RQ value is used to represent the fold change in quantitative real-time polymerase chain reaction detection. (**b**) Evaluation of MMP16 in four paired cancer tissues and their normal control by western blot. The results showed that there were higher MMP16 expression in cancer tissues than their controls’.

We first studied MMP16 mRNA expression in 20 paired cases. As anticipated, the MMP16 mRNA expression levels in cancer tissues were significantly higher than their paired adjacent normal mucosa (*P* < 0.001, Fig. 2a). Then, we test MMP16 expression in 4 paired cancer tissues and their normal tissues by western blot, the results showed that there were higher MMP16 in cancer tissues than their controls’ (Fig. 2b). We further studied MMP16 mRNA and protein expression in 10 CRC tissues and found the MMP16 mRNA expressions were consisted with their protein expression levels (Data not shown).

Then, as mentioned previously, we divided patients into high and low MMP16 expression subgroups according to median MMP16 expression value. High MMP16 expression was significantly correlated with N stage (*P* = 0.008) and lymphovascular invasion (*P* = 0.002) (Table 2).

**Figure 3:**
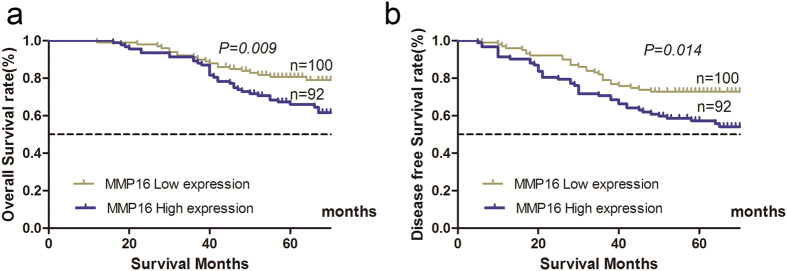
Influence of MMP16 expression patterns on overall survival (**a**) and disease free survival (**b**) by Kaplan-Meier analyses in the validation cohort.

The 5-year OS and DFS in high and low MMP16 groups were 66.0% and 80.6%, 54.3% and 72.8%, respectively, both of which have statistically significant difference (*P* < 0.05, Fig. 3a,b).

In a standardized way using Cox regression model, all factors that were statistically significant in the univariate were tested in multivariate Cox regression analysis for association with OS and DFS. Multivariate analysis demonstrated that high MMP16 expression level, poor tumor grade, and advanced T and N stage were independently associated with both OS and DFS (*P* < 0.05) (Tables 3 and 4).

### Silencing of MMP16 expression inhibits the migration and invasion of human colon cancer cells

**Figure 4:**
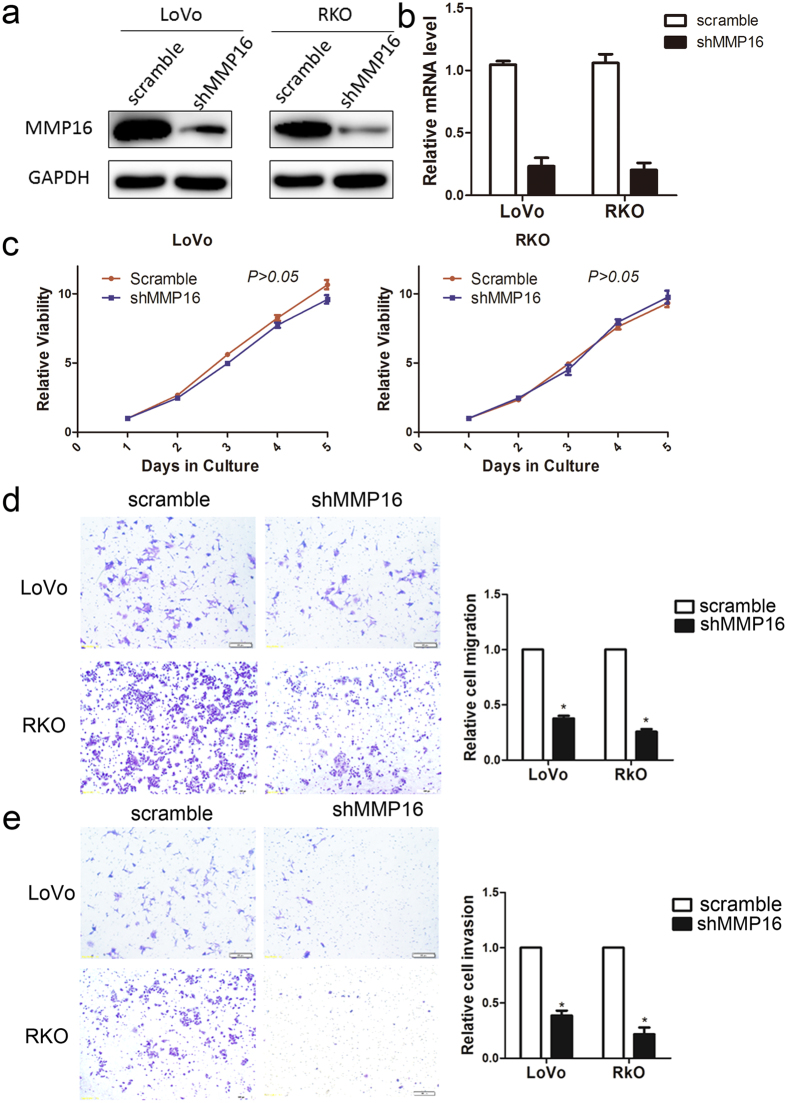
Influence of MMP16 in colon cancer cell proliferation and invasion. The MMP16 in LoVo and RKO cells after transfection of shRNA against MMP16 or scramble sequence was analyzed by Western blot (**a**) and RT-PCR (**b**). (**c**) Growth curves of LoVo and RKO cells with transfected shRNAs or scramble sequence. Cell growth was determined by CCK-8. Representative images were shown of migration (**d**) or invasion (**e**) of LoVo and RKO cells via transwells without or with matrigel, measured by direct counting of trespassing cells.

To determine the role of MMP16 in colon cancer cells viability and progression, we used lentivirus-mediated method to establishe stable MMP16-knockdown in LoVo and RKO cells, and the knockdown efficiency were v determined by RT-PCR and western blotting (Fig. 4a,b). CCK8 assay showed that no significantly different cell growth rates between MMP16-knockdown cells and their control cells was found. (*P* > 0.05, Fig. 4c). The effect of MMP16 on tumor cell migration and invasion were then measured by Transwell analysis without (migration) and with (invasion) matrigel, and the results demonstrated that there were significantly decreased in cell motility and invasion abilities in MMP16 knockdown cells, as compared with control cells. (*P* < 0.05, Fig. 4d,e).

## Discussion

Local recurrence and distant metastasis are suggested to be the key reasons for poor prognosis and cancer related death in tumor patients. Previous studies have shown that MMP16 is overexpressed in gastric cancer, glioma cancer and melanoma and has implications for tumor invasion and prognosis^10^,^11^,^12^,^13^. However, little is known regarding its expression pattern and clinical value in CRC. In this study, we first studied MMP16 expression in TCGA database and GSE39582 database, and found that its expression was correlated with poor OS. For TCGA and GSE39582 database lacks some important clinicopathological features (eg. lymphovascular invasion and perineural invasion) and therapy information (eg. radical resection or palliative resection), we then validated clinical value of MMP16 in in-house database and confirmed that high MMP16 expression in CRC was negatively correlated with both OS and DFS. Furthermore, functional study found knockdown of MMP16 expression could inhibit the migration and invasion of colon cancer cells.

MMP16 is one number of the important MMP family. MMP16 functions in activating pro-MMP2 (gelatinase A) into its active form as the zymogen is excreted out of the cell^14^. Therefore, activating MMP2 would be an indirect mechanism of determining the activity of MMP16^10^,^11^. The activated MMP2 can promote the migration and invasion of tumor cells^13^ by denaturing type IV collagen and partially degrading type I collagen and other ECM proteins in basement membrane^10^,^15^,^16^. Therefore, it is not surprising that high MMP16 expression promoted the invasion and metastasis abilities and led to poor survival outcomes in CRC. In the validation database, we demonstrated that MMP16 expression was significantly correlated with N stage and lymphovascular invasion, both of which were indicated of high invasive abilities of CRC. However, our results seems contradiction with the results from Moon *et al*. who demonstrated that the MMP16 promoter is frequently hypermethylated in CRC and that downregulation of MMP16 may increase cell migration in CRC^17^. Our results were first got from public available TCGA database and then validated in in-house database, which made our results more reliable and convincible. Xu *et al*. also confirmed MMP16 as oncogene in CRC^18^. MMP16 can promote the invasion and metastasis of melanoma cells by decreasing cell adhesion, inhibiting collagen alignment and inducing lymphatic invasion^12^. Overexpression of MMP16 can promote migration and invasion of gastric cancer cells and then cause worse long time survival in gastric cancer^10^. MMP16 is a downstream of β-catenin target gene in human gastric cancer, induction of the MMP16 protein expression is vital to the Wnt-mediated invasion and metastasis in gastric cancer cells^12^,^19^, all of which indicated that MMP16 acts as an oncogene by facilitating metastasis in solid tumor.

In summary, we combined analysis the public available database and in-house cohort firmly and demonstrated that overexpression of MMP16 was closely correlated with poor OS and DFS. Therefore, MMP16 can serve as an indicator of prognosis as well as a potential novel target for treatment in CRC patients.

## Materials and Methods

### Patients in TCGA and GSE39582 database

Gene expression (RNA-Seq) data and corresponding clinical data of CRC samples were retrieved from TCGA database ((https://genome-cancer.ucsc.edu/) and GSE39582 database (https://www.ncbi.nlm.nih.gov/geo/). All patients included in the study should be pathological diagnosed with adenocarcinoma, have no pretreatment, and with intact OS information. Patients who died within one months were excluded from this study. Patients who died with tumor at last follow-up were defined as the clinical endpoint for tumor specific survival. Follow-up was completed on Apr 27, 2016 in TCGA database on Feb 24, 2017 in GSE39582 database.

### Patients in the validation database

CRC specimens from patients who underwent intentionally curative surgical resection from January 2004 to December 2009 were obtained to validate the conclusions from TCGA database. Tumor tissues were histopathologically verified as adenocarcinoma and noncancerous tissues were confirmed as negative. Tissue fragments were immediately put in RNA-later and stored at −80 °C. Specimens and data were anonymized, and the need for ethical consent was obtained from the institutional ethics committee of The Affiliated Yancheng Hospital of Southeast University Medical College, Yancheng Third People’s Hospital. The methods were carried out in accordance with the approved guidelines. Written informed consent was obtained from all subjects. Inclusion criteria were patients with pathological confirmed colorectal adenocarcinomas, absence of distant metastasis (M0) at the time of surgery and without neoadjuvant chemotherapies. All patients were restaged according to 7^th^ edition TNM stage system. For OS analysis, patients who died at the last follow-up were defined as clinical endpoints. For analysis of DFS, tumor progression after surgical resection was the clinical endpoint, documented as either tumor recurrence or metastasis. Follow-up data were recorded by phone or medical records.

### Real-time PCR

MMP16 mRNA levels were analyzed using a real-time PCR assay. The total RNA from tissues was extracted using the TRIzol reagent (Invitrogen, Carlsbad, CA, USA) according to the manufacturer’s instructions, and reversely transcripted to cDNA with PrimeScript™ RT Master Mix (Perfect Real Time) kit (RR036A, Takara) based on the manufacturer’s instruction. RT-PCR was performed using the SYBR Green Master Mix (Roche, Mannheim, Germany) on an ABI 7900HT Fast Real-Time PCR System (Applied Biosystems, Foster City, CA, USA) in triplicate, and non-template controls were run for each assay under the same conditions. Primers were as follows: GAPDH-F, 5′-GCACCGTCAAGGCTGAGAAC-3′, GAPDH-R, 5′-TGGTGAAGACGCCAGTGGA-3′; MMP16-F, 5′-GGACAGAAATGGCAGCACAAGC-3′, MMP16-R, 5′-CATCAAAGGCACGGCGAATAGC-3′^10^.

The cycling conditions were as follows: initial denaturation for 10 min at 95 °C followed by 35 cycles of denaturation (15 s at 95 °C), annealing and elongation (30 s at 60 °C). The relative expression of MMP16 was calculated and normalized using the RQ value method relative to GAPDH.

### Western blotting

The MMP16 expression was assessed by western blotting analysis and samples were normalized to GAPDH. Total proteins were extracted from the cultured cells solubilized in lysis buffer (RIPA Lysis Buffer, Thermo Scientific Pierce). The protein were separated by sodium dodecyl sulfate-polyacrylamide gel electrophoresis and then transferred to polyvinylidene difluoride membranes (Bio-rad). The membranes were blocked within 5% Bovine Serum Albumin (BSA) at room temperature for 2 h and incubated overnight at 4 °C with primary anti-MMP16 (1:500, Abgent) and anti-GAPDH (1:5000, Santa Cruz), respectively. The membranes subsequently washed and incubated with appropriate secondary antibodies. After being incubated with ECL, the protein bands were visualized.

### Cell culture

The human CRC cell lines (LoVo and RKO) were originally purchased from the American Type Culture Collection (Manassas, VA, USA). Cells were cultured in DMEM medium (Invitrogen, Carlsbad, CA, USA) supplemented with 10% FBS (Invitrogen, Carlsbad, CA, USA) and 1% penicillin/streptomycin (Invitrogen).

### Stable transfection of colon cancer cells

Biologically active short hairpin RNAs (shRNA) were generated using the lentiviral expression vector pLKO.1-puro. The shRNA target sequence for human MMP16 was 5′-CGTGATGTGGATATAACCATT-3′. PLKO.1-scramble shRNA with limited homology with any known sequences in the human was used as a negative control. LoVo and RKO cells were transfected with the pLKO.1-shMMP16 expression vector or pLKO.1-scramble. The cells stably transfected were isolated using puromycin selection to obtain stable MMP16 knockdown cells.

### Cell proliferation assays

Cell proliferation Reagent Kit (CCK-8, Dojindo, Japan) was used to assess cell proliferation. Transfected cells were plated in each well of a 96-well plate and assessed every 24 h according to the manufacturer’s instructions. The cell viability of different groups at each measuring time point was compared.

### Cell migration and invasion assay

The migration and invasion ability of LoVo and RKO cells after different transfection was measured by Transwell assay (without or with matrigel). Approximately 10^5^ cells were seeded on the upper chamber of the transwell with 200 μl serum-free growth medium (10^5^ cells per well of 8.0 μm Pore Polycarbonate Membrane Insert). Complete medium containing 10% FBS was added to the lower chamber as a chemo-attractant. After 48 h of incubation at 37 °C, non-migratory cells on the upper surface of upper chamber were removed slightly by cotton swabs, and cells that migrated to the bottom of the membrane were fixed and stained. The number of invaded cells was counted under light microscope. To minimize the bias, five randomly selected fields with 200× magnification were counted, then the average number was calculated.

### Statistical Analysis

Two-tailed χ2 test was used to evaluate the expression difference between theclinicopathological features and MMP16 expression. The survival curves were estimated by Kaplan-Meier analysis, and *P* values were calculated by log rank test. Univariate Cox proportional hazards regressions were applied to estimate the individual hazard ratio (HR) for the DFS and OS. The HR with 95% confidence interval (CI) was measured to estimate the hazard risk of individual factors. All experiments were performed independently a minimum of three times. All *P* values were two-sided, and *P* < 0.05 was considered statistically significant. Statistical calculations were all performed using SPSS 17.0.

## Additional Information

**How to cite this article:** Wu, S. *et al*. High expression of matrix metalloproteinases 16 is associated with the aggressive malignant behavior and poor survival outcome in colorectal carcinoma. *Sci. Rep.* **7**, 46531; doi: 10.1038/srep46531 (2017).

**Publisher's note:** Springer Nature remains neutral with regard to jurisdictional claims in published maps and institutional affiliations.

## Figures and Tables

**Table 1:** Clinical characteristics of patients with colorectal cancer in the TCGA, GSE39582 and validation cohort.

Variable	TCGA		GSE39582		Validation Cohort	
	N	%	N	%	N	%
Sex						
Male	316	45.4	213	44.9	99	51.6
Female	263	54.6	261	55.1	93	48.4
Age	66	31–90	67	22–97	66	22–85
Grade						
G1	/	/	/	/	88	45.8
G2	/	/	/	/	77	40.1
G3	/	/	/	/	27	14.1
T stage						
T1/T2	115	19.9	52	11.0	46	24.0
T3/T4	462	79.8	418	88.2	146	76.0
TX	2	0.3	4	0.8	/	/
N stage						
N0	323	55.8	283	59.7	110	57.3
N1	144	24.9	108	22.8	55	28.6
N2	108	18.7	78	16.5	27	14.1
Nx	4	0.7	1	1.1	/	/
M stage						
M0	426	73.6	474	100	192	100
M1	83	14.3	/	/	/	/
Mx	70	12.1	/	/	/	/
Lymphovascular invasion						
Negative	318	54.9	/	/	168	87.5
Positive	201	34.7	/	/	24	12.5
Unknown	60	10.4	/	/	/	/
Perineural invasion						
Negative	/	/	/	/	164	85.4
Positive	/	/	/	/	28	14.6

**Table 2:** Association between MMP16 expression and clinic pathological factors in the validation cohort.

Variable	n	MMP16 Expression		χ^2^ Value	P value
		Low	High		
**Gender**				0.021	0.885
Male	99	50	49		
Female	93	46	47		
**Age**				0.209	0.647
≦60	65	31	34		
>60	127	65	62		
**T category**				0.457	0.499
T1/2	46	21	25		
T3/4	146	75	71		
**N stage**				9.546	0.008
N0	110	64	46		
N1	55	18	37		
N2	27	14	13		
**Pathological grading**				2.225	0.329
High	88	39	49		
Moderate	77	43	34		
Poor	27	14	13		
**Lymphovascular invasion**				9.733	0.002
Negative	168	91	77		
Positive	24	5	19		
**Perineural invasion**				0.669	0.413
Negative	164	84	80		
Positive	28	12	16		
**Ki67**				0.637	0.425
Negative	55	30	25		
Positive	137	66	71		

**Table 3:** Univariate and multivariate Cox proportional hazards analysis of MMP16 expression and overall survival for patients with colorectal cancer in the validation cohort.

Factor	Univariate analysis		Multivariate analysis	
	HR (95% CI)	P	HR (95% CI)	P
Gender	0.677 (0.403–1.136)	0.139		
Age	1.148 (0.659–2.000)	0.625		
Grade	1.909 (1.356–2.687)	**<0.001**	1.518 (1.045–2.205)	**0.028**
T category	7.152 (2.237–22.863)	**0.001**	4.273 (1.305–13.992)	**0.016**
N stage	3.765 (2.677–5.293)	**<0.001**	3.114 (2.069–4.685)	**<0.001**
Lymphovascular invasion	2.193 (1.182–4.069)	**0.013**	1.109 (0.585–2.103)	**0.750**
Perineural invasion	2.390 (1.329–4.299)	**0.004**	0.781 (0.406–1.503)	0.459
Tumor location	0.837 (0.491–1.427)	0.514		
MMP16	1.992 (1.168–3.395)	**0.011**	1.938 (1.129–3.372)	**0.038**

Abbreviation: CI, confidence interval; HR, hazard ratio.

Bold type indicates statistical significance.

**Table 4:** Univariate and multivariate Cox proportional hazards analysis of MMP16 expression and disease free survival for patients with colorectal cancer in the validation cohort.

Factor	Univariate analysis		Multivariate analysis	
	HR (95% CI)	P	HR (95% CI)	P
Gender	0.787 (0.488–1.268)	0.325		
Age	1.065 (0.641–1.770)	0.809		
T category	4.054 (1.752–9.377)	**0.001**	2.736 (1.155–6.483)	**0.022**
N stage	3.259 (2.377–4.469)	**<0.001**	2.711 (1.881–3.907)	**<0.001**
Grade	1.941 (1.411–2.671)	**<0.001**	1.650 (1.171–2.325)	**0.004**
Lymphovascular invasion	2.165 (1.202–3.900)	**0.010**	1.139 (0.616–2.106)	0.677
Perineural invasion	1.969 (1.108–3.491)	**0.021**	0.771 (0.412–1.443)	0.416
Tumor location	0.726 (0.439–1.200)	0.211		
MMP16	1.818 (1.118–2.955)	**0.022**	1.839 (1.122–3.016)	**0.023**

Abbreviation: CI, confidence interval; HR, hazard ratio.

Bold type indicates statistical significance.
